# Variation in the health outcomes associated with frailty among home care clients: relevance of caregiver distress and client sex

**DOI:** 10.1186/s12877-018-0899-8

**Published:** 2018-09-12

**Authors:** Colleen J. Maxwell, Michael A. Campitelli, Christina Diong, Luke Mondor, David B. Hogan, Joseph E. Amuah, Sarah Leslie, Dallas Seitz, Sudeep Gill, Kednapa Thavorn, Walter P. Wodchis, Andrea Gruneir, Gary Teare, Susan E. Bronskill

**Affiliations:** 1 Schools of Pharmacy and Public Health & Health Systems, University of Waterloo, 200 University Ave. W, Waterloo, ON N2L 3G1 Canada; 2 Institute for Clinical Evaluative Sciences, 2075 Bayview Ave, Toronto, ON M4N 3M5 Canada; 3 Health System Performance Research Network, Toronto, ON Canada; 4 Division of Geriatric Medicine, Department of Medicine, University of Calgary, HSC-3330 Hospital Drive NW, Calgary, AB T2N 4N1 Canada; 5 School of Epidemiology, Public Health & Preventive Medicine, University of Ottawa, 451 Smyth Road, Ottawa, ON K1H 8M5 Canada; 6 School of Public Health & Health Systems, University of Waterloo, 200 University Ave. W, Waterloo, ON N2L 3G1 Canada; 7 Division of Geriatric Psychiatry, Queen’s University & Providence Care Hospital, 752 King Street W, Kingston, ON K7L 4X3 Canada; 8 Department of Medicine, Queen’s University & Providence Care Hospital, 752 King Street W, Kingston, ON K7L 4X3 Canada; 9 Ottawa Hospital Research Institute, 501 Smyth Road, PO Box201B, Ottawa, ON K1H 8L6 Canada; 10 Institute of Health Policy Management & Evaluation, University of Toronto, 155 College Street, Toronto, ON M5T 3M6 Canada; 11 Department of Family Medicine, University of Alberta, 8440 112 St. NW, Edmonton, AB T6G 2R7 Canada; 12 Department of Community Health & Epidemiology, College of Medicine, University of Saskatchewan, Health Science Building, 107 Wiggins Rd, Saskatoon, SK S7N 5E5 Canada

**Keywords:** Home care, Frailty, Caregiver distress, Sex, Gender, Nursing home, Hospitalization, Mortality, Retrospective cohort

## Abstract

**Background:**

The identification of contextual factors that modify associations between client frailty and their health and service use outcomes is essential for informed home health care and policy planning. Our objective was to examine variation in the associations between frailty and select 1-year health outcomes by caregiver distress and client sex among community-residing older care recipients.

**Methods:**

We conducted a retrospective cohort study using linked population-based clinical and health administrative databases for all long-stay home care clients (*n* = 234,552) aged 66+ years assessed during April 2010–2013 in Ontario, Canada. Frailty was assessed using a previously validated 72-item frailty index (FI). Presence of caregiver distress was derived from clinical assessment items administered by trained home care assessors. Multivariable log-binomial regression models were used to examine variations in the associations between frailty and outcomes of interest (mortality, nursing home [NH] placement, all-cause and prolonged hospitalization) by caregiver distress, with further model stratification by client sex.

**Results:**

Frailty prevalence varied little by sex (19.3% women, 19.9% men) despite significant sex-differences in clients’ sociodemographic and health characteristics. In both sexes, frailty was significantly associated with all outcomes, particularly NH placement (RR = 3.84, 95%CI 3.75–3.93) and death (RR = 2.32, 95%CI 2.27–2.37), though risk ratios were greater for women. Caregiver distress was more common with increasing frailty and for male clients, and a significant independent predictor of NH placement and prolonged hospitalization in both sexes. The association between frailty and NH placement (but not other outcomes) varied by caregiver distress for both men and women (*p* < 0.001 interaction terms), showing a greater magnitude of association among clients without (vs. with) a distressed caregiver.

**Conclusions:**

As caregiver distress varies by client sex, represents a key driver of NH placement (even among relatively robust clients), and modifies the impact of other risk factors such as frailty, it should be routinely assessed. Further, sex-differences should be considered when developing and evaluating community-based services for older adults and their caregivers.

**Electronic supplementary material:**

The online version of this article (10.1186/s12877-018-0899-8) contains supplementary material, which is available to authorized users.

## Background

In Canada and the United States, the provision of in-home professional and supportive services for older adults is an important and growing part of the healthcare system [1, 2]. Proposals for healthcare reforms in both countries have called for a significant expansion of publicly funded home care, including the provision of additional support for family caregivers [3–5]. Underlying these reforms is the hope that enhanced community-based care may reduce both acute and nursing home facility admissions among vulnerable older adults [6–9]. As government payers face competing demands for their (limited) health budgets, identifying individuals at heightened risk for admission to facility-based care and thus, most likely to benefit from community-based services, has become a key priority [7, 10].

The identification of frailty offers a promising approach to risk stratification in this care setting [11, 12]. Frailty refers to a state of increased vulnerability to stressors arising from multi-system dysfunction leading to declines in homeostatic reserve and resiliency [12]. In previous work, we demonstrated both the feasibility and predictive validity of a frailty index measure derived from assessment data routinely collected on older home care recipients [10, 13]. In addition to predicting mortality and transitions to higher levels of care, this frailty measure was positively associated with the likelihood of caregiver distress. Intriguing questions emerging from this earlier work include the extent to which caregiver-related factors modify the associations between client frailty and relevant health outcomes such as hospitalization and NH placement and whether associations further vary by client sex. Given sex-related differences in health and the nature and availability of informal support [14–16], it is plausible that the impact of caregiver distress on frailty-related outcomes may differ by client sex.

Family and friends play a significant role in providing supportive care to vulnerable older adults in the community [4, 5, 17]. This role is viewed positively by many informal (or unpaid) caregivers who derive a sense of fulfillment from providing needed care or services [18–20]. At the same time, the increasingly complex nature of care recipient needs coupled with demanding caregiving roles and expectations can precipitate stress and poor outcomes for both care providers and recipients [21–24]. Consequently, the presence and extent of caregiver distress or burden requires consideration when assessing the larger impact of community-based care reforms for older adults. Caregiver distress has been shown to be an independent predictor of health outcomes and costs among community-dwelling older adults, particularly for those with dementia [25–28]. However, few large scale studies have examined its importance among the general population of older home care clients, and data are especially scarce on its role as an effect modifier of associations between frailty and health outcomes in this care setting [11].

Among the few studies that have examined moderators of frailty-related outcomes, the focus has largely been on the relevance of psychosocial resources (including perceived social support and wellbeing) and findings were inconclusive [29, 30]. There is also some preliminary data to suggest that more positive responses on a summary protection index (combining a diverse number of items reflecting an older adult’s socioeconomic status, lifestyle behaviours and environmental factors) may mitigate frailty-related mortality and health decline for robust or pre-frail older persons [31]. The relevance of such findings to more vulnerable older adults receiving home care is unknown. Further exploration of the impact of informal care characteristics on frailty-related outcomes among female and male home care recipients could inform policy development and resource allocation for this population [10, 32]. Additionally, illustrating effect modification by these contextual factors might enhance support for targeted and tailored community-based services for older adults [33].

Our primary objective was to examine the degree to which caregiver distress modifies the associations between frailty and select 1-year health outcomes among a population-based cohort of older home care clients. A secondary objective was to investigate variation in the role of this modifying factor between female and male clients.

## Methods

### Study design, setting and population

We conducted a retrospective cohort study of long-stay home care clients in Ontario, Canada using linked health administrative and clinical databases (see Additional file 1: Table S1). These databases were linked using encoded identifiers and analyzed at the Institute for Clinical Evaluative Sciences (ICES).

In Ontario, semi-annual assessments with the Resident Assessment Instrument for Home Care (RAI-HC) are provincially mandated for all clients receiving long-stay services (i.e., ≥ 60 days in a single episode). The RAI-HC is administered by trained staff and provides standardized validated data on clients’ sociodemographic characteristics, health conditions, physical and cognitive status, behaviors, and service use [34]. RAI-HC assessment data are included in the ICES repository. A summary of the organization and funding of Ontario home care at the time of this study is provided in Additional file 1: Table S1.

We identified all clients assessed between April 1, 2010 and March 31, 2013 (*n* = 296,964) and captured data from their earliest RAI-HC (index date). Given our focus on clients receiving services in the community and age requirements for data availability, we excluded those aged < 66 (*n* = 55,343; 18.6%) or > 105 (*n* = 24; 0.01%) years, receiving case management only (*n* = 4444; 1.5%), in a nursing home facility during the prior year (*n* = 2351; 0.8%), with data quality issues (*n* = 183; 0.06%), or a non-Ontario postal code (*n* = 67; 0.02%). The resulting sample included 234,552 clients.

This study was approved by the Research Ethics Board at Sunnybrook Health Sciences Centre and the University of Waterloo, Office of Research Ethics (ORE File #19950). Informed consent by participants was not required as this project was conducted under section 45 of Ontario’s Personal Health Information Protection Act and approved by ICES’ Privacy and Compliance Office.

### Frailty

Frailty was assessed with a validated 72-item frailty index (FI) based on items derived from the index RAI-HC assessment [10]. The FI was calculated as the proportion of accumulated to potential health deficits. Deficits included physical, cognitive, behavioural and psychosocial characteristics. As in previous work [10, 13], robust, pre-frail and frail clients were defined based on FI scores of < 0.2, 0.2–0.3, and > 0.3, respectively.

### Covariates

Clients’ age, sex, and date of death were determined from administrative data (Additional file 1: Table S1). Comorbidity was assessed with the Aggregated Diagnosis Groups (ADGs) derived using the Johns Hopkins Adjusted Clinical Group algorithms [35] and based on health service use in the two years prior to clients’ index date (The Johns Hopkins ACG® System, v10). Data regarding clients’ marital status, health conditions and caregiver characteristics were obtained from their index RAI-HC.

### Caregiver measures

Caregiver characteristics included the presence of a primary caregiver (and their relationship to, and living arrangement with, the client) and average hours of care per day for instrumental and basic activities of daily living (ADL) provided in the past week by family, friends and neighbours. Unfortunately, we did not have information on caregivers’ age or sex, though some assumptions could be made about both based on the information that was available to us from the RAI-HC (as described above). Caregiver distress was determined by a positive response to at least one the following two RAI-HC items: a caregiver reports or is perceived by the home care assessor to be unable to continue in caring activities (due to various reasons, e.g., a decline in health of caregiver, a lack of desire to continue, geographical inaccessibility, other competing family or work requirements or personal health issues) and/or caregiver reported feelings of distress, anger or depression. This is a standard and widely accepted measure of caregiver distress (burden) when using RAI-HC data and has been employed in numerous national and provincial health system quality reports [3, 4, 36, 37] and previous studies on home care in Canada [14, 38, 39].

### Outcomes

Outcomes assessed over the 1-year included death, nursing home (NH) placement, any (inpatient) hospitalization and prolonged hospital stay as derived from the administrative databases. In Ontario, the NH setting primarily encompasses long-stay residents requiring 24-h nursing and personal care and/or supervision. A prolonged hospitalization is defined for a patient who no longer requires the intensity of services provided in acute care but is unable to be discharged because adequate care is not available elsewhere (often because a NH bed is unavailable) [40].

### Analysis

Descriptive analyses examined the distribution of baseline characteristics and 1-year outcomes among the total sample and by frailty level. Associations between frailty and each of the binary outcomes were examined using log-binomial regression models to estimate risk ratios. Models were adjusted for age, sex and comorbidity to be consistent with previous work [10]. Associations between caregiver distress and the four outcomes were examined in separate models that were initially adjusted for age, sex and comorbidity and then also for frailty level.

To examine effect modification by caregiver distress, we derived a set of mutually exclusive variables to cross-classify clients by frailty and presence/absence of caregiver distress. For example, clients were classified into one of 6 categories defined by frailty level (robust, pre-frail, frail) and caregiver distress (yes, no) and four separate regression models (one for each outcome) were examined including this categorical variable while also adjusting for age, sex and comorbidity. This allows for direct estimation of the effect of frailty on outcomes at each level of the covariate and comparison of risk ratios within and between levels of the covariate. For this categorical variable, the reference group was selected to represent those expected to have the lowest risk (e.g., robust and caregiver not distressed). These analyses were then stratified by client sex.

We employed alternative modeling strategies (i.e., including an interaction term between frailty and caregiver distress) to derive tests of statistical significance for this interaction term. We were cautious when interpreting findings from these tests as even small differences would be expected to be highly significant given our sample size.

All analyses were conducted using SAS version 9.4 (SAS Institute Inc., Cary, NC).

## Results

The mean age of clients was 82 (±7.4) years, 65% were women and almost half were widowed (Additional file 1: Table S2). Most reported having a primary caregiver (98%) and for half of these clients, this caregiver lived in the same residence. The primary caregiver was most commonly a child or child-in-law (55.3%) followed by a spouse (31.2%), other relative (7.9%) and friend or neighbor (5.7%) [data not shown]. Clients received an average of 2.4 h of care from family or friends per day, and almost one quarter had a distressed caregiver. High levels of client morbidity were evident. Frailty was positively associated with client age, comorbidity level, informal care hours, and likelihood for the primary caregiver to live with the client and to be distressed.

Female clients were more likely to be older, widowed, and to not reside with their primary caregiver (Table 1). For women, relative to men, this caregiver was more likely to be a child or child-in-law (65.9% vs. 35.7%) as opposed to a spouse (19.5% vs. 52.6%) [data not shown]. Average hours of informal care received per day were higher for men than women as was the likelihood for a distressed caregiver (e.g., 51.8% of frail men and 39.8% of frail women had a distressed caregiver). Higher levels of comorbidity were observed among men although some conditions were more prevalent among women (e.g., hypertension, arthritis, and osteoporosis). Although statistically significant, there was little difference in frailty prevalence between women (19.3%) and men (19.9%).

**Table 1:** Baseline characteristics of long-stay home care clients in Ontario (2010–2013), by sex and frailty status (*n* = 234,552)

Characteristic	Women (*n* = 151,427)			Men (*n* = 83,125)		
	FI Frailty Status (n, column %)			FI Frailty Status (n, column %)		
	Robust (*n* = 69,709; 46.0%)	Pre-Frail (*n* = 52,549; 34.7%)	Frail (*n* = 29,169; 19.3%)	Robust (*n* = 38,967; 46.9%)	Pre-Frail (*n* = 27,606; 33.2%)	Frail (*n* = 16,552; 19.9%)
Age group						
66–74	12,154 (17.4)	8163 (15.5)	3800 (13.0)	9250 (23.7)	5354 (19.4)	2920 (17.6)
75–84	28,943 (41.5)	21,722 (41.3)	11,067 (37.9)	17,084 (43.8)	12,526 (45.4)	7350 (44.4)
85+	28,612 (41.0)	22,664 (43.1)	14,302 (49.0)	12,633 (32.4)	9726 (35.2)	6282 (38.0)
Mean age ± SD	82.1 ± 7.4	82.6 ± 7.3	83.6 ± 7.4	80.5 ± 7.5	81.3 ± 7.2	81.7 ± 7.2
Marital status						
Married	18,930 (27.2)	13,867 (26.4)	7647 (26.2)	23,450 (60.2)	17,254 (62.5)	10,916 (65.9)
Never married/other	3739 (5.4)	2256 (4.3)	1134 (3.9)	2601 (6.7)	1402 (5.1)	756 (4.6)
Widowed	42,506 (61.0)	32,987 (62.8)	18,817 (64.5)	9893 (25.4)	7038 (25.5)	3935 (23.8)
Separated/Divorced	4534 (6.5)	3439 (6.5)	1571 (5.4)	3023 (7.8)	1912 (6.9)	945 (5.7)
Primary Caregiver						
No primary caregiver	1857 (2.7)	926 (1.8)	372 (1.3)	1276 (3.3)	515 (1.9)	235 (1.4)
Yes, does not live with	40,437 (58.0)	28,388 (54.0)	14,430 (49.5)	13,799 (35.4)	8868 (32.1)	4962 (30.0)
Yes, lives with	27,415 (39.3)	23,235 (44.2)	14,367 (49.3)	23,892 (61.3)	18,223 (66.0)	11,355 (68.6)
Average hours of informal care per day,^a^						
mean ± SD	1.60 ± 1.80	2.39 ± 2.59	3.25 ± 3.83	2.01 ± 2.07	3.09 ± 2.96	3.82 ± 4.09
Caregiver is distressed						
No	62,992 (90.4)	40,566 (77.2)	17,557 (60.2)	32,833 (84.3)	17,999 (65.2)	7985 (48.2)
Yes	6717 (9.6)	11,983 (22.8)	11,612 (39.8)	6134 (15.7)	9607 (34.8)	8567 (51.8)
# ADG comorbidity categories						
0–5	11,766 (16.9)	7554 (14.4)	3743 (12.8)	5089 (13.1)	3082 (11.2)	1485 (9.0)
6–9	23,646 (33.9)	16,163 (30.8)	8142 (27.9)	11,862 (30.4)	7742 (28.0)	4023 (24.3)
10+	34,297 (49.2)	28,832 (54.9)	17,284 (59.3)	22,016 (56.5)	16,782 (60.8)	11,044 (66.7)
Most prevalent diagnoses						
Hypertension	41,260 (59.2)	35,755 (68.0)	20,623 (70.7)	20,160 (51.7)	16,614 (60.2)	10,660 (64.4)
Arthritis	37,575 (53.9)	32,496 (61.8)	18,075 (62.0)	13,382 (34.3)	11,834 (42.9)	7418 (44.8)
Diabetes	13,281 (19.1)	14,177 (27.0)	8876 (30.4)	10,229 (26.3)	9120 (33.0)	6142 (37.1)
Coronary artery disease	11,900 (17.1)	14,032 (26.7)	8859 (30.4)	9357 (24.0)	9346 (33.9)	6486 (39.2)
Osteoporosis	19,175 (27.5)	17,665 (33.6)	10,829 (37.1)	2355 (6.0)	2379 (8.6)	1663 (10.0)

Abbreviations: *FI* = frailty index, *SD* = standard deviation, *ADG* = Aggregated Diagnosis Groups

^a^Hours of care for instrumental and basic activities of daily living in past week by family, friends and neighbours

Note: for all comparisons across frailty level, *p* < 0.001

### Associations between frailty, caregiver distress and outcomes

During the 1-year follow-up, 18% of clients died, 17% were admitted to a NH, 42% experienced at least 1 hospitalization and 14% had a prolonged hospital stay. The proportion of clients experiencing each outcome increased significantly with frailty level (Additional file 1: Table S3). The distribution of outcomes by sex**,** caregiver distress, and frailty are presented in Additional file 1: Tables S4 and S5. With the exception of NH placement, all outcomes were more common among men.

Following adjustment for age, sex and comorbidity, higher frailty levels were significantly associated with all outcomes, most notably NH placement and death (Table 2, base models). Further stratification by sex showed similar findings although for all outcomes except prolonged hospitalization, risk ratios associated with frailty were higher among women than men. Overall and for both sexes, including frailty and caregiver distress in the same model (Table 2, full models) had little effect on frailty-related risk estimates for most outcomes with the exception of NH placement where risk estimates were somewhat attenuated.

**Table 2:** Associations between client frailty status, caregiver distress and risk of health outcomes during 1 year follow-up, overall and by client sex

	Outcomes at 1 year							
	Death		NH Placement		Any Hospitalization		Prolonged Hospitalization^a^	
	Base Models^b^ Risk Ratio (95% CI)	Full Models^c^ Risk Ratio (95% CI)	Base Models^b^ Risk Ratio (95% CI)	Full Models^c^ Risk Ratio (95% CI)	Base Models^b^ Risk Ratio (95% CI)	Full Models^c^ Risk Ratio (95% CI)	Base Models^b^ Risk Ratio (95% CI)	Full Models^c^ Risk Ratio (95% CI)
OVERALL								
Client Frailty (FI)								
Robust (ref)								
Pre-frail	1.45 (1.42–1.48)	1.46 (1.43–1.49)	2.20 (2.15–2.26)	2.08 (2.03–2.13)	1.17 (1.16–1.19)	1.17 (1.16–1.19)	1.42 (1.39–1.45)	1.40 (1.37–1.43)
Frail	2.32 (2.27–2.37)	2.34 (2.29–2.40)	3.84 (3.75–3.93)	3.39 (3.31–3.48)	1.22 (1.20–1.23)	1.22 (1.20–1.23)	1.51 (1.47–1.55)	1.47 (1.43–1.51)
Caregiver Distress								
No (ref)								
Yes	1.21 (1.19–1.24)	0.97 (0.95–0.98)	1.95 (1.92–1.99)	1.40 (1.37–1.42)	1.06 (1.05–1.07)	1.00 (0.99–1.01)	1.23 (1.20–1.25)	1.10 (1.08–1.13)
WOMEN								
Client Frailty (FI)								
Robust (ref)								
Pre-frail	1.51 (1.47–1.56)	1.52 (1.47–1.56)	2.25 (2.18–2.32)	2.13 (2.07–2.20)	1.20 (1.19–1.22)	1.20 (1.18–1.22)	1.43 (1.39–1.48)	1.41 (1.37–1.45)
Frail	2.61 (2.53–2.69)	2.62 (2.54–2.70)	4.06 (3.94–4.17)	3.58 (3.47–3.69)	1.26 (1.24–1.28)	1.25 (1.23–1.27)	1.52 (1.47–1.57)	1.47 (1.42–1.52)
Caregiver Distress								
No (ref)								
Yes	1.29 (1.25–1.32)	0.99 (0.96–1.02)	2.02 (1.97–2.06)	1.41 (1.38–1.45)	1.08 (1.07–1.10)	1.02 (1.00–1.03)	1.25 (1.21–1.28)	1.12 (1.08–1.15)
MEN								
Client Frailty (FI)								
Robust (ref)								
Pre-frail	1.40 (1.35–1.44)	1.41 (1.37–1.45)	2.14 (2.05–2.22)	2.00 (1.92–2.08)	1.14 (1.12–1.16)	1.14 (1.12–1.16)	1.40 (1.35–1.45)	1.38 (1.33–1.43)
Frail	2.07 (2.00–2.13)	2.10 (2.04–2.17)	3.48 (3.35–3.62)	3.07 (2.95–3.20)	1.17 (1.15–1.19)	1.18 (1.15–1.20)	1.50 (1.44–1.56)	1.46 (1.40–1.52)
Caregiver Distress								
No (ref)								
Yes	1.16 (1.13–1.19)	0.95 (0.93–0.98)	1.85 (1.80–1.91)	1.37 (1.32–1.41)	1.04 (1.02–1.05)	0.99 (0.97–1.00)	1.21 (1.17–1.24)	1.08 (1.05–1.12)

Abbreviations: *FI* = frailty index, *NH* = nursing home, *ADG* = Aggregated Diagnosis Groups

^a^Excludes clients hospitalized *without* a prolonged bed stay (overall sample, *n* = 66,764)

^b^Risk estimates also adjusted for age, sex (overall models only) and ADG comorbidity, *p* < 0.001 for all estimates

^c^Risk estimates from models including age, sex (overall models only), ADG comorbidity, FI frailty status and caregiver distress, *p* < 0.001 for all estimates except:

- caregiver distress relationship to any hospitalization for overall model (*p* = 0.8096); among women (*p* = 0.0415); among men (*p* = 0.1765)

- caregiver distress relationship to death among women (*p* = 0.4072)

In similarly adjusted base models, caregiver distress was significantly associated with client risk for all outcomes, with stronger associations observed for NH placement and weaker associations noted for other outcomes, especially any hospitalization (Table 2, base models). Similar findings were observed for models further stratified by sex. Following further adjustment for client frailty (Table 2, full models), caregiver distress remained significantly associated with NH placement and prolonged hospitalization only.

### Modification of frailty-outcome associations by caregiver distress

The associations between frailty and death, hospitalization and prolonged hospitalization were not modified by caregiver distress (Table 3). For example, the risk of prolonged hospitalization for frail vs. robust clients was 1.42 among those without a distressed caregiver and 1.49 (1.68/1.13) among those with a distressed caregiver. For some outcomes, interaction terms were statistically significant (i.e., for any hospitalization, *p* = 0.002 for frail*caregiver distress; for prolonged hospitalization, *p* = 0.002 for pre-frail*caregiver distress) largely reflecting the sample size rather than meaningful variation.

**Table 3:** Associations between client frailty status - caregiver distress categorical variable and risk of health outcomes during 1 year follow-up, overall and by client sex

	Outcomes at 1 year, risk ratio (95% CI)			
	Death	NH Placement	Any Hospitalization	Prolonged Hospitalization^a^
Client Frailty (FI) and Caregiver Distress, Overall^b^				
CG not distressed & Robust	1	1	1	1
CG not distressed & Pre-Frail	1.47 (1.43, 1.50)	2.20 (2.14, 2.27)	1.18 (1.16, 1.19)	1.43 (1.40, 1.47)
CG not distressed & Frail	2.36 (2.30, 2.42)	4.00 (3.88, 4.11)	1.20 (1.18, 1.21)	1.42 (1.38, 1.47)
CG distressed & Robust	0.99 (0.95, 1.04)	2.10 (2.01, 2.20)	0.99 (0.96, 1.01)	1.13 (1.08, 1.19)
CG distressed & Pre-Frail	1.41 (1.37, 1.46)	3.26 (3.16, 3.37)	1.16 (1.14, 1.18)	1.48 (1.43, 1.53)
CG distressed & Frail	2.26 (2.20, 2.32)	4.79 (4.65, 4.93)	1.24 (1.22, 1.26)	1.68 (1.62, 1.73)
Ratio [Fr v Robust] CG not distressed	2.36	4.00	1.20	1.42
Ratio (Fr v Robust] CG distressed	2.28	2.28	1.25	1.49
Client Frailty (FI) and Caregiver Distress, Women^b^				
CG not distressed & Robust	1	1	1	1
CG not distressed & Pre-Frail	1.52 (1.47, 1.58)	2.22 (2.15, 2.30)	1.20 (1.18, 1.22)	1.43 (1.39, 1.48)
CG not distressed & Frail	2.64 (2.55, 2.73)	4.15 (4.01, 4.29)	1.23 (1.20, 1.25)	1.43 (1.37, 1.49)
CG distressed & Robust	1.03 (0.95, 1.11)	2.16 (2.04, 2.30)	0.98 (0.95, 1.02)	1.13 (1.06, 1.21)
CG distressed & Pre-Frail	1.50 (1.42, 1.57)	3.42 (3.29, 3.57)	1.20 (1.17, 1.23)	1.51 (1.44, 1.58)
CG distressed & Frail	2.59 (2.49, 2.69)	5.05 (4.88, 5.24)	1.30 (1.27, 1.33)	1.71 (1.64, 1.79)
Ratio [Fr v Robust] CG not distressed	2.64	4.15	1.23	1.43
Ratio (Fr v Robust] CG distressed	2.51	2.34	1.33	1.51
Client Frailty (FI) and Caregiver Distress, Men^b^				
CG not distressed & Robust	1	1	1	1
CG not distressed & Pre-Frail	1.41 (1.36, 1.46)	2.18 (2.08, 2.29)	1.15 (1.13, 1.17)	1.43 (1.37, 1.49)
CG not distressed & Frail	2.09 (2.01, 2.17)	3.69 (3.51, 3.88)	1.15 (1.13, 1.18)	1.41 (1.34, 1.49)
CG distressed & Robust	0.95 (0.89, 1.01)	1.98 (1.85, 2.12)	0.98 (0.95, 1.01)	1.13 (1.06, 1.21)
CG distressed & Pre-Frail	1.33 (1.27, 1.39)	3.00 (2.85, 3.16)	1.12 (1.09, 1.14)	1.44 (1.37, 1.52)
CG distressed & Frail	2.01 (1.94, 2.09)	4.34 (4.14, 4.56)	1.18 (1.15, 1.21)	1.64 (1.56, 1.72)
Ratio [Fr v Robust] CG not distressed	2.09	3.69	1.15	1.41
Ratio (Fr v Robust] CG distressed	2.12	2.19	1.20	1.45

Abbreviations: *FI* = frailty index, *NH* = nursing home, *CG* = caregiver;

^a^Excludes clients hospitalized *without* a prolonged bed stay (overall sample, *n* = 66,764)

^b^Models adjusted for age, sex (overall models only) and ADG comorbidity; all estimates *p* < 0.001 except:

- caregiver distressed - robust for death: overall (*p* = 0.8345); women (*p* = 0.4499); men (*p* = 0.0871)

- caregiver distressed - robust for any hospitalization: overall (*p* = 0.3063); women (*p* = 0.4029); men (*p* = 0.1932)

**Fig. 1:**
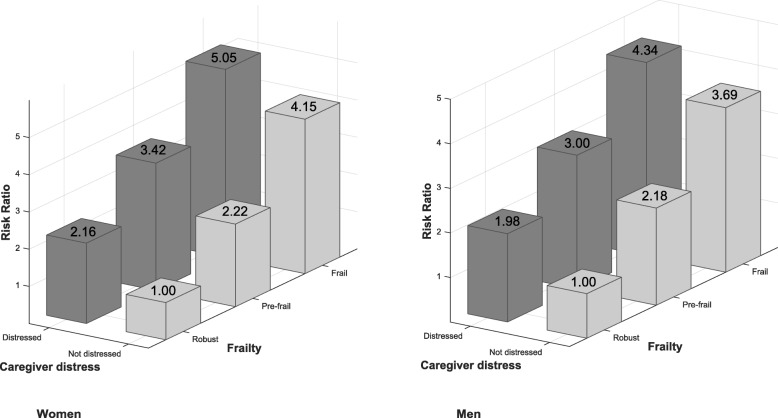
Variation in the association between frailty status and risk of nursing home placement during 1 year follow-up according to caregiver distress and client sex among long-stay home care clients in Ontario (2010–2013)

The association between frailty and NH placement was modified by caregiver distress, both overall and when stratified by client sex (Table 3; Fig. 1). Specifically, while increasing frailty was associated with increased risk of placement among those with and without a distressed caregiver, the magnitude of the association was greater among clients *without* than with a distressed caregiver (*p* < 0.001, all interaction terms). For example, the risk of placement for frail vs. robust clients was 4 fold among those *without* a distressed caregiver, and 2.3 fold (4.79/2.10) among those with a distressed caregiver. Relative to the reference group (client robust and caregiver not distressed), the combination of frailty and a distressed caregiver was associated with a 5 fold increased risk of placement for women and a 4 fold increased risk for men.

## Discussion

Building on our earlier work [10], higher frailty levels were significantly and independently associated with all health outcomes examined among both women and men. Risk estimates were strongest for NH placement and death and higher for women than men for most outcomes. Interestingly, despite significant sex-related differences in clients’ sociodemographic and health characteristics, frailty prevalence did not vary by client sex with approximately 1 in 5 women and men classified as frail. However, caregiver distress, which increased significantly with increasing frailty and hours of informal care, was considerably more common among male than female clients.

After adjusting for covariates (including frailty), clients with a distressed caregiver were 40% more likely to be placed in a NH and 10% more likely to experience a prolonged hospitalization and these findings were comparable for female and male clients. The presence of caregiver distress did not appear to modify the associations between frailty and death or hospitalization events. There was, however, evidence of important effect modification by caregiver distress for the association between frailty and NH placement. Overall, and for women and men separately, the impact of frailty on placement was of a greater magnitude among those without (vs. with) a distressed caregiver.

There is general agreement that caregiver burden is an important predictor of institutionalization, particularly among older adults with dementia [25–27]. Our findings demonstrate that this association extends to the wider population of older home care clients not selected for cognitive impairment. Although we lacked specific information on caregiver sex, previous literature [27, 41–44] and our descriptive findings suggest that much of this informal care (and associated distress) falls disproportionately on female caregivers. For example, among older male clients, the primary caregiver was more commonly a spouse whereas among older female clients, the primary caregiver was more likely to be a child or child-in-law. As a prolonged hospitalization often precedes a transition to NH, it is not surprising that caregiver distress was also associated with this outcome [45]. Importantly clients with a distressed caregiver and who were also frail showed the highest risks for both NH placement and prolonged hospitalization, relative to the lowest risk group.

At first glance, the observation that the association between frailty and NH placement was greater among clients *without* than *with* a distressed caregiver seems counterintuitive. However, this largely reflects the significant contribution of caregiver distress to placement among less frail clients. For example, robust clients with (vs. without) a distressed caregiver were twice as likely to be admitted to a NH.

We observed higher risk estimates for death, NH placement and hospitalization associated with frailty among women relative to men. For death and hospitalization, this may reflect higher baseline risks for these outcomes among men. This would not explain the greater impact of frailty on placement among women as baseline risk was similar for men and women. The increased risk among women may be related to their increased likelihood to be widowed and to differences in their caregiver characteristics relative to male clients. There is some literature suggesting that female caregivers [25] and those who live with the care recipient [16], a scenario more likely for male clients in our study, may be less likely to pursue NH placement. This pattern may persist even with increasing client frailty level, leading to higher levels of distress for (female) caregivers as they strive to keep their loved one at home [42–44].

Strengths of our study include the population-based sample of older home care clients and availability of comprehensive clinical, functional, and psychosocial measures derived from the linked databases. Our analyses employed a frailty measure previously validated for this population [10]. Our focus on community-dwelling care recipients adds to the existing literature on the relevance of caregiver distress to health outcomes among older adults.

Limitations include the absence of detailed data on the informal caregivers, including their sex and the nature of, and amount of time spent on, caregiving activities. Similarly, for both informal and formal care providers, we did not have access to qualitative aspects of their relationships with clients which may be important to caregiver distress and client outcomes [18, 19, 44]. This would include both positive and negative aspects of caregiving, which may show different patterns for female and male caregivers and between spouses and children [20, 41]. Despite these noted concerns, it is important to investigate the modifying effect of caregiver distress (as assessed with the RAI-HC) on client outcomes given its availability for population-based analyses. With the widespread implementation of the RAI-HC instrument in Canada and beyond, demonstrating the utility of these caregiver items for informing community-based practice and policy decisions is an important research priority. It should also be noted that our data do not allow us to comment on the relevance of caregiver support (or burden) to vulnerable older adults in the community not receiving formal care [43]. Additional research is required to more fully understand what underlies these observed associations and the potential impact of interventions designed to either minimize caregiver distress or delay frailty progression.

## Conclusions

Our findings highlight the extensive involvement of unpaid caregivers in providing assistance to home care clients and the impact of this care on their health and on care recipient outcomes. On average, caregivers provided almost 2.5 h of care per day, which increased to 3.5 h for clients who were frail. Among frail clients, almost half had distressed caregivers, an estimate double that noted for the total sample. With continued shifts from institutional to community-based care and increased levels of clinical complexity and frailty among older home care recipients, the prevalence of caregiver distress is likely to increase [46]. Our findings that caregiver distress is a significant driver of NH placement, even among relatively robust clients, and modifies the impact of other risk factors such as client frailty, demonstrate the importance of implementing routine assessments of caregiver burden and family-centered interventions as core elements of optimal community-based care [33, 46–48]. Despite ongoing calls for increased publicly-funded services and support for family caregivers, our findings and those of others [1–4, 36] point to lingering concerns of significant unmet needs among both care recipients and their caregivers.

As we also observed important sex-differences in caregiver relationships and likelihood for distress and in the magnitude of associations between frailty and NH placement, it is essential that both caregiver and care recipient sex be considered in research and planning of services for vulnerable home care populations [49, 50]. More extensive and innovative changes in relevant home and social care policies and funding arrangements will be needed to permit greater flexibility in how support services are packaged and targeted to meet the unique needs of care recipient and caregiver dyads.

## Additional file

Additional file 1 **Table S1.** (Description of Ontario Home Care and Clinical/Health Administrative Databases); **Table S2.** (Baseline characteristics of long-stay home care clients in Ontario (2010–2013), by frailty status (*n* = 234,552)); **Table S3.** (Proportion of long-stay home care clients who experienced each outcome^a^ during 1 year follow-up, by frailty status); **Table S4.** (Proportion of long-stay home care clients who experienced each outcome^a^ during 1 year follow-up, by sex and frailty status); **Table S5.** (Proportion of long-stay home care clients who experienced each outcome^a^ during 1 year follow-up, by presence of caregiver distress and frailty status). (DOCX 33 kb)

## Abbreviations

ADGs: Aggregated Diagnosis Groups
CG: Caregiver
FI: Frailty index
NH: Nursing home
RAI-HC: Resident assessment instrument for home care
SD: Standard deviation
