# Supplementary material for: Variation in the health outcomes associated with frailty among home care clients: relevance of caregiver distress and client sex
Source: BMC Geriatr. 2018 Sep 12;18:211. doi: 10.1186/s12877-018-0899-8 (PMC6134755; doi:10.1186/s12877-018-0899-8)
Supplement: Supplementary file 1 — Table S1. (Description of Ontario Home Care and Clinical/Health Administrative Databases); Table S2. (Baseline characteristics of long-stay home care clients in Ontario (2010–2013), by frailty status (n = 234,552)); Table S3. (Proportion of long-stay home care clients who experienced each outcomea during 1 year follow-up, by frailty status); Table S4. (Proportion of long-stay home care clients who experienced each outcomea during 1 year follow-up, by sex and frailty status); Table S5. (Proportion of long-stay home care clients who experienced each outcomea during 1 year follow-up, by presence of caregiver distress and frailty status). (DOCX 33 kb) [file 12877_2018_899_MOESM1_ESM.docx]

**Additional file 1:**

**Table S1: Description of Ontario Home Care and Clinical/Health Administrative Databases**

**Table S2: Baseline characteristics of long-stay home care clients in Ontario (2010-2013), by frailty status (n=234,552)**

**Table S3: Proportion of long-stay home care clients who experienced each outcome^a^ during 1 year follow-up, by frailty status**

**Table S4: Proportion of long-stay home care clients who experienced each outcome^a^ during 1 year follow-up, by sex and frailty status**

**Table S5: Proportion of long-stay home care clients who experienced each outcome^a^ during 1 year follow-up, by presence of caregiver distress and frailty status**

**Table S1: Description of Ontario Home Care and Clinical/Health Administrative Databases**

Ontario is Canada’s largest province with approximately 13.5 million residents. Almost all are covered by a government-funded universal health insurance program that pays for all medically necessary hospital and physician services and includes coverage of prescription drugs for persons aged 65+ years or receiving social assistance. The province is divided into 14 Local Health Integration Networks (LHINs) that are responsible for the planning and funding of local health services. At the time of this study, provincial home care services were centrally managed and coordinated through 14 Community Care Access Centres (CCACs) (aligned with each LHIN) that operated as a single point of entry for Ontarians requiring home care services.^1,6,26^ [see also, <http://www.homecareontario.ca/home-care-services/facts-figures/publiclyfundedhomecare>] The CCAC coordinates delivery of provincially-funded community-based services and facilitates the nursing home admission process for those assessed as eligible for this level of care. Case managers within each CCAC determine eligibility for services and coordinate service provision. Services may include homemaking, transportation, personal care, nursing care, physiotherapy, occupational and speech-language therapy. As a result of regionalization (and service maximums defined for public funding), there may be variation in the type and amount of services provided across the 14 CCACs. Home care is provided on either a short- or long-stay basis, where the latter refers to clients requiring care for 60 or more days in a single episode. For all long-stay clients, routine assessment with the Resident Assessment Instrument for Home Care (RAI-HC) is provincially mandated. All services provided from the public health insurance system are recorded in databases (see brief summary of databases below) and later linked deterministically using unique encoded identifiers and analyzed at the Institute for Clinical Evaluative Sciences (ICES) in Toronto.

| **Database** | **Description** |
| --- | --- |
| Registered Persons Database (RPDB) | A population-based registry maintained by the Ontario Ministry of Health and Long-Term Care, used to derive clients’ sociodemographic characteristics (age, sex, postal code) and date of death; |
| Canadian Institute for Health Information Discharge Abstract Database (CIHI-DAD) | Capturing data on all hospital discharges from all acute care centres in the province of Ontario, used to derive selected diagnoses, comorbidity and health service use measures; |
| National Ambulatory Care Reporting System (NACRS) | Includes information on hospital- and community-based ambulatory care services provided in the province of Ontario, used to capture data on emergency department (ED) use; |
| Ontario Health Insurance Plan (OHIP) Claims Database | Capturing data on all physician billing for services provided to Ontario residents, used to derive selected diagnoses, comorbidity and health service use measures; |
| Ontario Drug Benefit (ODB)  Database | Capturing all prescription medication claims for individuals covered under the provincial drug program and used to derive selected diagnoses and drug measures; |
| Home Care Reporting System (HCRS) | Includes data collected with the Resident Assessment Instrument for Home Care (RAI-HC), a standardized mandated assessment administered by trained home care staff at regular intervals to all long-stay, publicly funded home care clients in Ontario. Data include clinical, functional and resource information and are used for care planning and quality monitoring purposes; |
| Continuing Care Reporting System (CCRS) | Includes detailed clinical assessment and placement data on individuals receiving continuing care services (including long-term care) in Ontario, used to determine NH admission and stays; |

**Table S2 Baseline characteristics of long-stay home care clients in Ontario (2010-2013), by frailty status (n=234,552)**

| **Characteristic** | **Overall N (%)** | **Frailty (FI) Status (n, column %)** | | |
| --- | --- | --- | --- | --- |
|  |  | **Robust (n=108,676; 46.3%)** | **Pre-Frail**  **(n=80,155; 34.2%)** | **Frail**  **(n=45,721; 19.5%)** |
| Mean age ± SD  Age group  66-74  75-84  85+ | 82.0 ± 7.4  41,641 (17.8)  98,692 (42.1)  94,219 (40.2) | 81.5 ± 7.5  21,404 (19.7)  46,027 (42.4)  41,245 (38.0) | 82.1 ± 7.3  13,517 (16.9)  34,248 (42.7)  32,390 (40.4) | 82.9 ± 7.4  6,720 (14.7)  18,417 (40.3)  20,584 (45.0) |
| Sex  Women  Men | 151,427 (64.6) 83,125 (35.4) | 69,709 (64.1)  38,967 (35.9) | 52,549 (65.6)  27,606 (34.4) | 29,169 (63.8)  16,552 (36.2) |
| Marital status  Married  Never married/other  Widowed  Separated/Divorced | 92,064 (39.3)  11,888 (5.1)  115,176 (49.1)  15,424 (6.6) | 42,380 (39.0)  6,340 (5.8)  52,399 (48.2)  7,557 (7.0) | 31,121 (38.8)  3,658 (4.6)  40,025 (49.9)  5,351 (6.7) | 18,563 (40.6)  1,890 (4.1)  22,752 (49.8)  2,516 (5.5) |
| Primary Caregiver  No primary caregiver  Yes, does not live with  Yes, lives with | 5,181 (2.2)  110,884 (47.3)  118,487 (50.5) | 3,133 (2.9)  54,236 (49.9)  51,307 (47.2) | 1,441 (1.8)  37,256 (46.5)  41,458 (51.7) | 607 (1.3)  19,392 (42.4)  25,722 (56.3) |
| Average hours of informal care per day,* mean ± SD | 2.38 ± 2.78 | 1.74 ± 1.91 | 2.63 ± 2.74 | 3.45 ± 3.93 |
| Caregiver is distressed  No  Yes | 179,932 (76.7)  54,620 (23.3) | 95,825 (88.2)  12,851 (11.8) | 58,565 (73.1)  21,590 (26.9) | 25,542 (55.9)  20,179 (44.1) |
| No. ADG comorbidity categories  0-5  6-9  10+ | 32,719 (13.9)  71,578 (30.5)  130,255 (55.5) | 16,855 (15.5)  35,508 (32.7)  56,313 (51.8) | 10,636 (13.3)  23,905 (29.8)  45,614 (56.9) | 5,228 (11.4)  12,165 (26.6)  28,328 (62.0) |
| Most prevalent diagnoses  Hypertension  Arthritis  Diabetes  Coronary Heart Disease  Osteoporosis | 145,072 (61.9)  120,780 (51.5)  61,825 (26.4)  59,980 (25.6)  54,066 (23.1) | 61,420 (56.5)  50,957 (46.9)  23,510 (21.6)  21,257 (19.6)  21,530 (19.8) | 52,369 (65.3)  44,330 (55.3)  23,297 (29.1)  23,378 (29.2)  20,044 (25.0) | 31,283 (68.4)  25,493 (55.8)  15,018 (32.8)  15,345 (33.6)  12,492 (27.3) |

**Abbreviations:** FI=frailty index; SD=standard deviation; ADG=Aggregated Diagnosis Groups

* Hours of care for instrumental and basic activities of daily living provided in past week by family, friends and neighbours

**Note**: for all comparisons across frailty level, p<0.001

**Table S3: Proportion of long-stay home care clients who experienced each outcome^a^ during 1 year follow-up, by frailty status**

| **Characteristic** | **Overall N (%)** | **FI Frailty Status (n, column %)** | | |
| --- | --- | --- | --- | --- |
|  |  | **Robust** | **Pre-Frail** | **Frail** |
| **Number of individuals** | 234,552 | 108,676 | 80,155 | 45,721 |
| **Death** | 41,044 (17.5) | 13,212 (12.2) | 14,307 (17.8) | 13,525 (29.6) |
| **NH placement** | 40,144 (17.1) | 9,372 (8.6) | 15,308 (19.1) | 15,464 (33.8) |
| **Any hospitalization**  **Length of stay (days), mean ± SD** | 98,385 (41.9)  14.1 ± 23.4 | 40,850 (37.6)  12.7 ± 20.5 | 35,876 (44.8)  14.8 ± 24.0 | 21,659 (47.4)  15.9 ± 27.2 |
| **Prolonged hospitalization** | 31,621 (13.5) | 11,927 (11.0) | 12,205 (15.2) | 7,489 (16.4) |
| **Prolonged hospitalization vs. no hospitalization^b^** | 31,621 (18.8) | 11,927 (15.0) | 12,205 (21.6) | 7,489 (23.7) |

**Abbreviations:** FI=frailty index; NH=nursing home; SD=standard deviation

^a^ Outcomes are not mutually exclusive; p<0.001 for comparisons of all outcomes across frailty level

^b^ Denominator excludes clients hospitalized *without* a prolonged bed stay (n=66,764); relevant sample sizes are: n=167,788 (overall cohort); n=79,753 (robust); n=56,484 (pre-frail); n=31,551 (frail)

**Table S4: Proportion of long-stay home care clients who experienced each outcome^a^ during 1 year follow-up, by sex and frailty status**

|  | **Women** | | | **Men** | | | **Total** |
| --- | --- | --- | --- | --- | --- | --- | --- |
|  | **Frailty Status (FI)** | | | **Frailty Status (FI)** | | |  |
|  | **Robust** | **Pre-Frail** | **Frail** | **Robust** | **Pre-Frail** | **Frail** |  |
| **Number of Individuals** | 69,709 | 52,549 | 29,169 | 38,967 | 27,606 | 16,552 | **234,552** |
| **Death** | 6,569 (9.4%) | 7,627 (14.5%) | 7,483 (25.7%) | 6,643 (17.0%) | 6,680 (24.2%) | 6,042 (36.5%) | 41,044 (17.5%) |
| **NH placement** | 5,875 (8.4%) | 9,978 (19.0%) | 10,241 (35.1%) | 3,497 (9.0%) | 5,330 (19.3%) | 5,223 (31.6%) | 40,144 (17.1%) |
| **Any hospitalization** | 23,704 (34.0%) | 21,855 (41.6%) | 12,855 (44.1%) | 17,146 (44.0%) | 14,021 (50.8%) | 8,804 (53.2%) | 98,385 (41.9%) |
| **Prolonged hospitalization** | 7,123 (10.2%) | 7,472 (14.2%) | 4,426 (15.2%) | 4,804 (12.3%) | 4,733 (17.1%) | 3,063 (18.5%) | 31,621 (13.5%) |
| **Number of Individuals^b^** | 53,128 | 38,166 | 20,740 | 26,625 | 18,318 | 10,811 | **167,788** |
| **Prolonged hospitalization vs. no hospitalization^b^** | 7,123 (13.4%) | 7,472 (19.6%) | 4,426 (21.3%) | 4,804 (18.0%) | 4,733 (25.8%) | 3,063 (28.3%) | 31,621 (18.8%) |

**Abbreviations:** FI=frailty index; NH=nursing home

^a^ Outcomes are not mutually exclusive

^b^ Excludes clients hospitalized *without* a prolonged bed stay (n=66,764 for total cohort)

Note: Adjusting for age, comorbidity and frailty, the risk estimates for men compared with women were 1.61 (95% CI 1.59-1.64) for death, 1.02 (95% CI 1.00-1.03) for NH placement, 1.22 (95% CI 1.21-1.23) for hospitalization and 1.33 (95% CI 1.30-1.35) for prolonged hospitalization.

**Table S5: Proportion of long-stay home care clients who experienced each outcome^a^ during 1 year follow-up, by presence of caregiver distress and frailty status**

|  | **Primary caregiver not distressed** | | | **Primary caregiver distressed** | | | **Total** |
| --- | --- | --- | --- | --- | --- | --- | --- |
|  | **Frailty Status (FI)** | | | **Frailty Status (FI)** | | |  |
|  | **Robust** | **Pre-Frail** | **Frail** | **Robust** | **Pre-Frail** | **Frail** |  |
| **Number of Individuals** | 95,825 | 58,565 | 25,542 | 12,851 | 21,590 | 20,179 | **234,552** |
| **Death** | 11,588 (12.1%) | 10,418 (17.8%) | 7,569 (29.6%) | 1,624 (12.6%) | 3,889 (18.0%) | 5,956 (29.5%) | 41,044 (17.5%) |
| **NH placement** | 7,301 (7.6%) | 9,884 (16.9%) | 7,986 (31.3%) | 2,071 (16.1%) | 5,424 (25.1%) | 7,478 (37.1%) | 40,144 (17.1%) |
| **Any hospitalization** | 36,040 (37.6%) | 26,278 (44.9%) | 11,793 (46.2%) | 4,810 (37.4%) | 9,598 (44.5%) | 9,866 (48.9%) | 98,385 (41.9%) |
| **Prolonged hospitalization** | 10,282 (10.7%) | 8,748 (14.9%) | 3,821 (15.0%) | 1,645 (12.8%) | 3,457 (16.0%) | 3,668 (18.2%) | 31,621 (13.5%) |
| **Number of Individuals^b^** | 70,067 | 41,035 | 17,570 | 9,686 | 15,449 | 13,981 | **167,788** |
| **Prolonged hospitalization vs. no hospitalization^b^** | 10,282 (14.7%) | 8,748 (21.3%) | 3,821 (21.7%) | 1,645 (17.0%) | 3,457 (22.4%) | 3,668 (26.2%) | 31,621 (18.8%) |

**Abbreviations:** FI=frailty index; NH=nursing home

^a^ Outcomes are not mutually exclusive

^b^ Excludes clients hospitalized *without* a prolonged bed stay (n=66,764 for total cohort)
